# Effectiveness of horticultural therapy in aged people with depression: A systematic review and meta-analysis

**DOI:** 10.3389/fpubh.2023.1142456

**Published:** 2023-03-08

**Authors:** Meijing Xu, Shan Lu, Jianjiao Liu, Feng Xu

**Affiliations:** ^1^College of Horticulture, China Agricultural University, Beijing, China; ^2^Faculty of Architecture, Building and Planning, University of Melbourne, Melbourne, VIC, Australia

**Keywords:** horticultural therapy, aged people, depression, meta-analysis, systematic review

## Abstract

**Background:**

Depression, an increasing global crisis, has affected many people's daily life, especially for older adults. Horticultural therapy has been widely used in non-pharmacological treatment for patients with depression, with a body of studies demonstrating its therapeutic effects. However, a lack of systematic reviews and meta-analyses makes it difficult to get a holistic picture of this research field.

**Objectives:**

We aimed to evaluate the reliability of the previous studies and the effectiveness of horticultural therapy (including the intervention of environmental settings, activities, and duration) on older adults with depression.

**Methods:**

This systematic review was conducted under the Preferred Reporting Items for Systematic Review and Meta-Analysis Protocols (PRISMA) guidelines. We searched relevant studies in multiple databases, and the original search was finished on 25 September 2022. We included studies using randomized controlled trials (RCTs) or quasi-experimental designs.

**Results:**

We yielded a total of 7,366 studies and finally included 13 which involved 698 aged people with depression. Results from meta-analysis indicated significant effects of horticultural therapy on reducing depressive symptoms for the older adults. Besides, we found different outcomes among various horticultural interventions (such as environmental setting, activities, and duration). Depression reduction was more effective in care-providing settings than in community settings; participatory activities were more effective in reducing depression than observational activities; intervention of 4–8 weeks might represent the optimal course of treatment compared to interventions more than 8 weeks in duration.

**Conclusion:**

We came up with a comprehensive set of recommendations based on the meta-analysis: aged people in care-providing settings with depression could get the most benefit from horticultural therapy by participating in participatory activities for 4–8 weeks.

**Systematic review registration:**

https://www.crd.york.ac.uk/prospero/display_record.php?ID=CRD42022363134, identifier CRD42022363134.

## 1. Introduction

Population aging has become a severe societal problem worldwide and accordingly the older adults' overall quality of life should be given adequate attention (1). World Health Organization defined health as a state of complete physical, mental, and social wellbeing, thus mental health and wellbeing are as important as, if not more important than, physical health (2). The older adults are more likely to experience various types of psychosocial stress due to reduced social contact, living alone, and health concerns, all of which exacerbate their emotions of loneliness and helplessness, increasing the possibility of depression (3). According to the report named *Depression: A Global Crisis* released by the United Nations (4), depression was ranked as the third leading cause of the global burden of disease in 2004 and will move into the first place by 2030. Around 3.7–34.8% of the older adults in the general population suffered from depression up to 2019 (5).

Depression in the twilight life was an affect-related disease marked by considerable and persistent depression, as well as changes in mind, behavior, and somatic symptoms, normally with long-lasting effects and a high relapse rate (6, 7). Depression might cause great suffering for both patients and caregivers, and lead to impaired functioning in patients' daily life. The major effects of depression included physical and psychological discomfort, worsening of pre-existing diseases, cognitive impairment, greater financial loads on families, and increased suicide rates (8, 9). For aged people, depression was a common cause of morbidity and disability and was commonly associated with Alzheimer's disease (8, 10–13). Depression in the aged made up a significant proportion of national health care budgets and there is a strong need for low-cost and effective strategies to treat depression in aged people.

Pharmacological interventions have been considered the primary treatment for depression for a long time (14, 15). However, medication may have side effects, such as increased risks of cardiovascular disease and metabolic syndrome (16). As a result, non-pharmacological methods–using physical and non-chemical methods rather than medications, are becoming increasingly popular in the treatment for aged people with depression (17, 18). Non-pharmacological treatments are significantly more cost-effective and feasible, and they have been increasingly employed as a first-line treatment before pharmacological treatment (19–22).

Horticultural therapy was regularly adopted as a non-pharmacological treatment for depression (23–28). Horticultural therapy is defined as the participation in horticultural activities facilitated by registered horticultural therapists to achieve specific goals within an established treatment, rehabilitation, or vocational plan (29). In recent decades, researchers and health practitioners have come to realize the potential health benefits derived from these activities, and empirical evidence for the effectiveness of horticulture therapy on the older adults with depression has received increasing attention (30–32). Previous systematic reviews have supported the effectiveness of horticultural therapy on various populations involving healthy people (33), patients with dementia (34), peoples with schizophrenia (35), people with depressive symptoms (36), etc. When participating in horticultural activities, older adults could improve their quality of life by changing monotonous life patterns, diverting attention from harmful emotions (such as anxiety, sadness) and illness, enhancing self-confidence and self-esteem, and preventing depression (37–40). It is also worth noting that the horticultural interventions of environmental settings, activities, and duration have significant impacts on the outcomes of depression-reduction effects (41–43).

Overall, studies have done abundant work on unveiling the depression-reduction effects of horticultural therapy. However, it is difficult for researchers to obtain a holistic picture of this topic due to the lack of a systematic review on the older adults with depression (measured or/and clinical confirmed) and the best dose of a “prescription pill”–the interventions of horticultural therapy, including the environmental settings, activities, and duration. Meta-analysis is a fundamental approach for evidence-based medical research which helps to resolve inconsistencies among studies by aggregating their results, in order to draw the most definitive conclusions from a statistical perspective (44). Therefore, we utilized meta-analysis to measure the effectiveness of horticulture therapy on aged people suffering from depression. We aim to evaluate existing studies, provide a comprehensive understanding of the effects of horticultural therapy on older adults with depression, and provide suggestions and inspirations for future research.

## 2. Methods

### 2.1. Search strategy

This systematic review was conducted under the Preferred Reporting Items for Systematic Review and Meta-Analysis (PRISMA) guidelines (45), which are presented in Supplementary Table 1. This study protocol was registered in the International Prospective Register of Systematic Reviews (PROSPERO) with registration number CRD42022363134. We searched relevant studies in several databases including PubMed, Embase, The Cochrane Library, Medline, CINAHL, PsycINFO, Web of Science, Scopus, ProQuest, and four Chinese databases–China National Knowledge Infrastructure (CNKI), Wanfang Data, VIP Data, and Chinese BioMedical Literature Database (CBM), with restrictions on the published year from database inception to 25 September 2022. We used the following common keywords: “aged/ elderly/‘old people'/older”, “Melancholia^*^/‘Depressive Disorder'/depress^*^”, and “horticult^*^/garden^*^/farm^*^”, and the Supplementary Tables 2–14 listed full search strings applicable to all 13 databases. The search strategy for Chinese databases was as follows: “(SU=‘老人 (old people)' OR SU=‘老年(aged)' OR SU=‘老龄(older)' OR SU=‘长者(older adults)')AND (SU=‘抑郁(depression)' OR SU=‘忧郁(depressive symptoms)') AND (SU=‘花园(garden)' OR SU=‘园艺(horticulture)' OR SU=‘农(farm)').”

### 2.2. Inclusion criteria and exclusion criteria

We included studies by the following criteria: (i) Population: older adults aged 60 or above with measured or clinically confirmed depression; (ii) Intervention: horticultural therapy; (iii) Comparison: conventional work training and other non-pharmacological treatments; (iiii) Outcomes: the score of depression; (iiiii) Study design: randomized controlled trials (RCTs) and quasi-experimental studies. Literature of which the original research data is missing or the full text is unavailable, duplicate publications of research data or literature, literature not in English or Chinese, and conference papers or dissertations were excluded.

### 2.3. Study selection and data extraction

All studies were imported into EndNote X9. We screened the titles and abstracts to exclude irrelevant literature, followed by a full-text assessment. The data extracts included: author, year of publication, country, age, gender, sample size, environmental settings (confined care-providing settings such as nursing homes, health center and hospital/unconfined community settings), performer, intervention method (activities, frequency, duration), follow-up, and outcome indicators, as well as mean and standard deviation (SD) score of depression. Other test parameters (e.g., Confidence Interval (CI), *p* and *F*) were used to calculate SD when it was not available. We also contacted the authors when an effect size could not be calculated. Two investigators screened the literature and extracted information independently, and a third investigator was involved when there were disagreements.

### 2.4. Quality assessment

The Cochrane Collaboration's risk of bias tool (46) was used to evaluate the risk of bias in the included RCT studies. The risk of bias assessment consisted of seven essential sources of bias: random sequence generation, allocation concealment, blinding of subjects and investigators, blinding of outcome assessors, incomplete outcome data, selective reporting of results and bias from other sources, with the expressions “low risk,” “high risk” and “unclear” representing the outcomes of the assessment.

The Joanna Briggs Institute (JBI) Critical appraisal tool were used to evaluate the risk of bias in the included quasi-experimental studies. The quality assessment consisted of nine items, covering causality of study variables, baseline, control, measurement of outcome indicators, and data analysis, with the expressions “Yes,” “No,” “Unclear” and “Not applicable” representing the outcomes of the assessment. Two investigators independently assessed all included studies, and when disagreements arose, two investigators discussed with each other or referred to the third investigator for consultation.

### 2.5. Statistical analysis

RevMan 5.3 software and Stata version 15.1 software were used for data processing, analyzing, and graphical plotting. The effect sizes were determined using standardized mean differences (SMDs) because the data included were continuous data measured on different scales. The results were aggregated with 95% CIs. The I^2^ test was used to assess the heterogeneity of intervention effects among studies, with 25, 50, and 75% being considered as low, moderate, and high degrees of heterogeneity respectively. We used the random-effects model when there was substantial between-study heterogeneity (I^2^ > 50%); otherwise a fixed effects model was used. If heterogeneity differences were too large, then subgroup analyses based on the study design and sensitivity analyses were performed to explore potential sources of heterogeneity and evaluate the robustness of the results. Furthermore, the funnel plot (if there were sufficient studies) and Egger's test (*p* > 0.05 (two-tailed) indicates no publication bias) were performed to assess the risk of publication bias.

## 3. Results

### 3.1. Search outcomes

**Figure 1:**
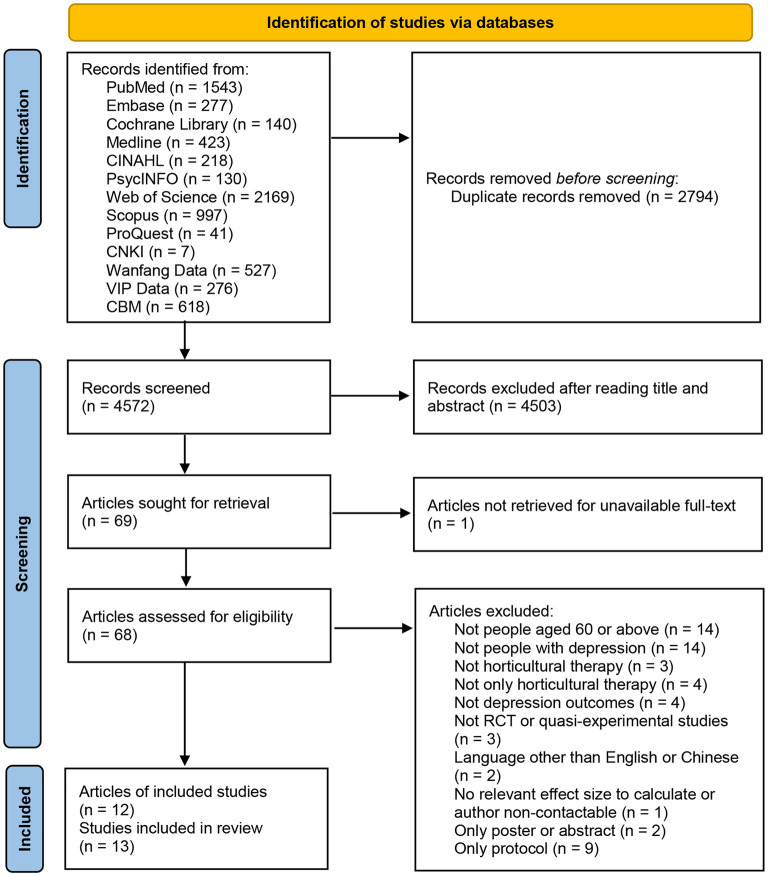
Flow diagram for the systematic review process.

The literature screening process and results are presented in Figure 1 according to the PRISMA flow diagram (47). We yielded a total of 7,366 articles; we eliminated 2,794 articles due to duplication, 4.503 due to irrelevant titles and abstracts, and 57 articles due to the failure to meet the criteria or unavailable full text (e.g., not people aged 60 or above, not people with depression, or not horticultural therapy). Twelve articles reporting 13 studies were finally included in this meta-analysis since two studies were from the same article (48). Six of which were RCTs and seven were quasi-experimental studies.

### 3.2. Study characteristics

We included a total of 13 studies that were published between 2010 and 2022, involving 698 older people with depression. Supplementary Table 15 shows the details of these studies. A majority of studies were performed in China (*n* = 6), followed by Japan (*n* = 3), Korea (*n* = 3) and Poland (*n* = 1). Most retrieved studies (*n* = 8; 61.5%) used the 15-item Geriatric Depression Scale (GDS-15), with remaining five studies using the 30-item Geriatric Depression Scale (GDS-30). The levels of depression were mild to moderate, with no aged people suffering from major depression in the reported studies.

The studies involved in the final analysis varied greatly in sample size. For example, Chu et al. (37) recruited 150 participants, and Wang and Jiang (49) recruited 126 participants, with six studies including < 30 participants. Nine studies had both male and female participants, while study from McCaffrey et al. (50) did not describe the gender composition of the participants, and studies from Kim (48) and Szczepanska et al. (51) had only female participants. All of the aged people involved in the studies had normal cognitive function, except for Kim (48). Some studies also included specific groups of people who, in addition to the depression they exhibited, may also have mild dementia (48), or memory problem (12). Ten studies involved interventions of horticultural therapy that were guided by professionals such as researchers (37, 50, 52–54), therapist (51), horticultural therapist (48, 55), and experts (12), while three studies did not mention any information about the performer (49, 56, 57).

Regarding the environmental settings, four studies were conducted in community (12, 50, 54, 56), three in nursing home (37, 55, 57), four in special care facilities (48, 51, 52), and one in hospital (49). Almost all studies were conducted in realistic environment, with two studies conducted using Virtual-Reality (VR) technology (51, 53). Seven studies were quasi-experimental studies, of which five were before-after design and two used a parallel design. Six RCTs were also parallel-designed studies. For parallel-designed studies, three studies adopted art therapy (50), sports care (49) and a blank (52), respectively as control, while the other two studies adopted leisure or education activities or usual care as a control. In terms of horticultural therapy, 11 studies selected participatory activities such as planting and harvest, and two involved observational activities such as walking in the green areas (50, 54). The course of treatment–duration of interventions ranged from 4 weeks (51) to 36 weeks (54), with each session lasting ~90 min on average (ranging from 20 to 120 minutes). The frequency of the interventions mainly included activities on daily, weekly, and monthly bases (12, 49, 54).

### 3.3. Methodological quality

**Figure 2:**
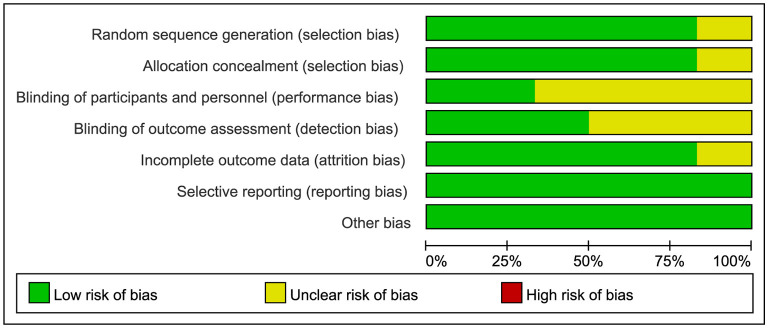
Risk of bias graph of RCTs.

**Figure 3:**
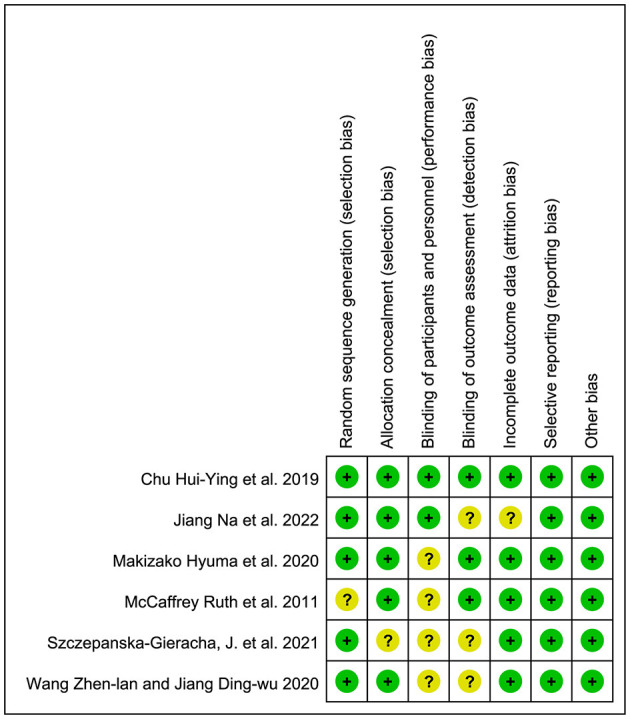
Summary of the risk of bias of RCTs.

Figures 2, 3 shows the evaluations of the risk of bias in six RCTs. Most of the studies were assessed as low-risk, with some studies having a loss to follow-up bias. One study mentioned “random” but did not explain the specific method they adopted, and the remaining studies described how the random sequence was generated, i.e., computer randomization. Five studies used central random assignment or sealed opaque envelopes, while others did not describe the concealment of allocation. Two studies were blinded to subjects. Three studies were blinded to the outcome measures. Only one study had incomplete outcome data, but the number and reasons for loss to follow-up bias were similar in the control and experimental groups. Selective bias was not found in all studies, and there was no other bias. Table 1 shows the detailed results of the risk of bias in seven quasi-experimental studies. In general, no risk of bias was found in the quasi-experimental studies included, according to the JBI critical appraisal checklist.

**Table 1:** Risk of bias of quasi-experimental studies.

**Included study**	**1**	**2**	**3**	**4**	**5**	**6**	**7**	**8**	**9**
Chen and Ji (57)	Yes	Yes	Yes	Yes	Yes	Yes	Yes	Yes	Yes
Yun et al. (56)	Yes	Yes	Yes	Yes	Yes	Yes	Yes	Yes	Yes
Kim (41)	Yes	Yes	Yes	Yes	Yes	Yes	Yes	Yes	Yes
Kim et al. (48)	Yes	Yes	Yes	Yes	Yes	Yes	Yes	Yes	Yes
Kim et al. (52)	Yes	Yes	Yes	Yes	Yes	Yes	Yes	Yes	Yes
Lin et al. (53)	Yes	Yes	Yes	Yes	Yes	Yes	Yes	Yes	Yes

1. Is it clear in the study what is the “cause” and what is the “effect” (i.e., there is no confusion about which variable comes first)? 2. Were the participants included in any comparisons similar? 3. Were the participants included in any comparisons receiving similar treatment/care, other than the exposure or intervention of interest? 4. Was there a control group? 5. Were there multiple measurements of the outcome both pre and post the intervention/exposure? 6. Was follow-up complete and, if not, were differences between groups in terms of their follow-up adequately described and analyzed? 7. Were the outcomes of participants included in any comparisons measured in the same way? 8. Were outcomes measured in a reliable way? 9. Was appropriate statistical analysis used?

### 3.4. Meta-analysis results

**Figure 4:**
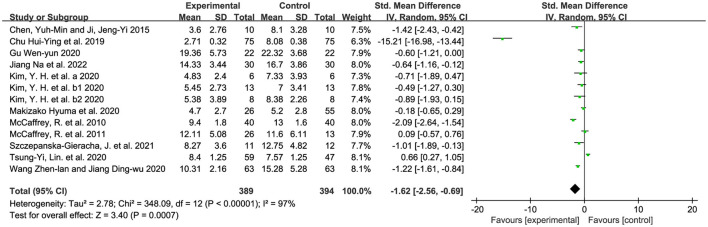
Effects on the depression.

The retrieved studies used the GDS-15 and the GDS-30 to measure the depression-reduction effects, and SMD was used because of the non-uniform standard. We used the random-effects model, accounting for diversity in horticultural interventions across studies (58). As shown in Figure 4, a significantly positive difference was found in the impact of horticultural therapy [SMD = −1.62, 95% CI (−2.56, −0.69), *p* = 0.0007], though with high heterogeneity detected in the pooled analysis (*p* < 0.00001, I^2^ = 97%). A sensitivity analysis was performed by removing the study included one by one and a significant decreased heterogeneity (I^2^ = 88%) was detected after removing the study from Chu et al. (37) [SMD = −0.69, 95% CI (−1.19, −0.19), *p* = 0.007]. Nevertheless, this analysis still suggested that horticultural therapy positively reduced depression for the older adults, indicating a robust effect.

### 3.5. Results of subgroup-analysis

Subgroup analysis was conducted according to the interventions of horticultural therapy including environmental settings, activities, and duration, to analyze the heterogeneity within the subgroups.

#### 3.5.1. Intervention of horticultural therapy–environmental settings

**Figure 5:**
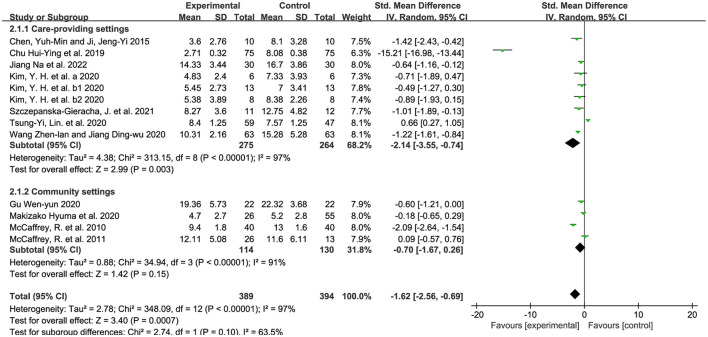
Effects on the symptoms in different environmental settings.

We divided the intervention settings into care-providing settings and community settings based on the well-accepted ways of aged care around the world (59, 60). SMD was used because of the non-uniform standard. We used the random-effects model because of the existence of substantial heterogeneity (*p* < 0.00001, I^2^ = 97%). In Figure 5, the depression-reduction effects were significant in care-providing settings [SMD = −2.14, 95% CI (−3.55, −0.74), *p* = 0.003] while not found in community settings [SMD = −0.70, 95% CI (−1.67, 0.26), *p* = 0.15]. We removed all the studies one by one and no changes were detected in sensitivity analyses.

#### 3.5.2. Intervention of horticultural therapy–activities

**Figure 6:**
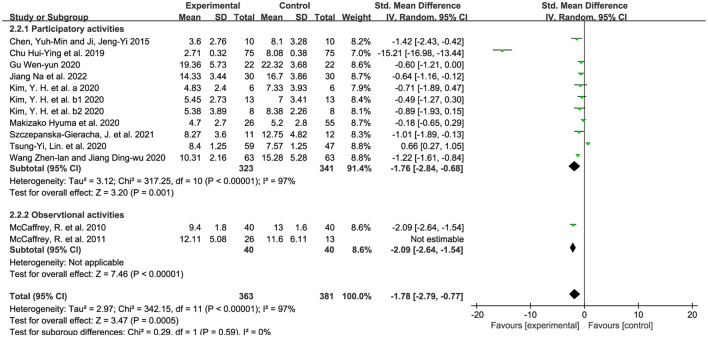
Effects on the symptoms in different activities.

The majority of the horticultural activities included planting and walking, and we categorized them into participatory and observational types according to previous studies (61, 62). SMD was used as well because of the non-uniform standard. We used the random-effects model because of the existence of substantial heterogeneity (*p* < 0.00001, I^2^ = 97%). Figure 6 demonstrates significant effects of horticultural therapy in the participatory activities [SMD = −1.76, 95% CI (−2.84, −0.68), *p* = 0.001] on the score of GDS, whereas the outcomes were not significant in the observational activities [SMD = −1.01, 95% CI (−3.15, 1.13), *p* = 0.36]. We removed all the studies one by one and the result became opposite in sensitivity analyses when removing the study from McCaffrey et al. (50) [SMD = 2.09, 95% CI [−2.64, −1.54], *p* < 0.00001) in observational activities.

#### 3.5.3. Intervention of horticultural therapy–duration

**Figure 7:**
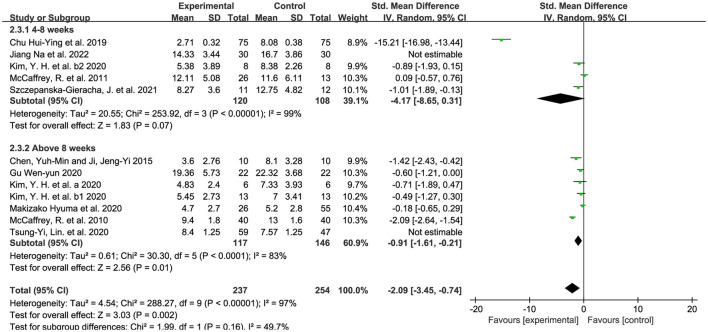
Effects on the symptoms in different duration.

We divided the intervention duration into 4–8 weeks and more than 8 weeks, due to the majority of the included studies and previous studies being 8 weeks. We used the random-effects model because of the existence of substantial heterogeneity (*p* < 0.00001, I^2^ = 97%), with SMD used. As shown in Figure 7, the result indicated significant effects of horticultural therapy in the duration of 8 weeks and below [SMD = −3.40, 95% CI (−6.36, −0.44), *p* = 0.02] and significant differences were detected in sensitivity analyses when removing the study from Jiang et al. (55) [SMD = −4.17, 95% CI (−8.65, 0.31), *p* = 0.07]. No difference was found between experimental group and control group in the duration of more than 8 weeks [SMD = −0.67, 95% CI (−1.47, 0.13), *p* = 0.10] and the result became opposite in sensitivity analyses when removing the study from Lin et al. (53) [SMD = −0.91, 95% CI (−1.61, −0.21), *p* = 0.01].

### 3.6. Publication bias

**Figure 8:**
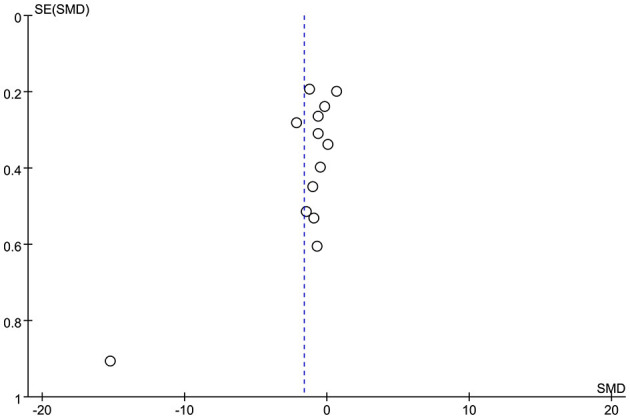
Funnel plot of included studies.

The funnel plot shows that the included studies are mainly clustered at the top and sparsely distributed at the bottom (Figure 8). They have symmetrical trends and are evenly dispersed on both sides, suggesting there was no publication bias. The conclusion was further supported by Egger's test, which found no evidence of publication bias (*p* = 0.062).

## 4. Discussion

This study provided a quantitative synthesis of the evidence supporting the positive effects of horticulture therapy on older adults people with depression. Previous research has shown that horticultural therapy could improve participants' emotions (63), reduce their stress level (64, 65), build confidence and self-esteem (66, 67), and improve their motor skills. During the activities, patients were willing to discuss and share their positive experiences with peers, which could strengthen their social interaction skills and increase their sense of community (68). This study provides a comprehensive prospect over the non-pharmacological prescription–horticultural therapy for aged people with depression, and we have identified several important attributes of horticultural therapy from subgroup analysis.

### 4.1. Care-providing settings were identified as more effective than community settings

Environmental settings could have essential impacts on effective treatment, in consistence with prior findings (69–72). Our results supported the therapeutic effects of horticultural activities in care-providing settings rather than those in community settings. We supposed that the reasons could be as follows: (i) Patients in care-providing settings might have a stronger desire to be cured compared to patients in community settings, which positively support their recovery from depression. (ii) The care-providing environments include a variety of specialized care environments such as hospitals and nursing homes, and featured a more professional medical and nursing team compared to communities. Thus, horticultural therapy was more likely to be long-termed and regular in care-providing settings.

The therapeutic effect to a large extent depends on the healing design for the environment and the degree of proximity to nature. Therefore, we appeal for more studies characterizing the healing environment in a clear and detailed way to shed light on the quantitative impact of the environment on healing outcomes.

### 4.2. Participatory activities were identified as more effective in depression reduction

The effectiveness of horticulture therapy was also influenced by the types of activities. We found that participatory activities were more beneficial in reducing depression than observational activities. People involved in participatory activities were more physically engaged than those in observational activities (73), which could strengthen their body functions and improve their physical condition. Simultaneously, participating in horticulture activities allowed individuals to enjoy their horticultural task meanwhile removed their negative emotions (74). Furthermore, aged people with depression may experience a variety of tactile sensations such as the temperature, hardness, and texture of different plants in participatory activities, which may stimulate the intuitive and motor parts of the cerebral cortex, resulting in a sense of comfort and enjoyment (75–77).

Our findings also suggest that when one study (50) on the efficiency of horticultural therapy in observational activities was excluded, the results turned out to be the contrary. This shows that the current evidence of the effectiveness of observational activities is not robust. The results of this test represent more of an indication than evidence, though many studies have shown that simply being exposed to nature could have a long-lasting and deep impact on health, both physically and psychologically (78–82). Moreover, given the limited studies on the observational activities, more well-designed studies are needed to evaluate the effectiveness.

### 4.3. Intervention of 4–8 weeks might represent the optimal course of treatment

Studies reported in this review varied in the duration and frequency of horticultural therapy. According to our results, the optimal intervention for older adults with depression was identified to be 4–8 weeks (basically once each week). The probable explanation might be that participants can completely participate in the life cycle of a certain plant. A longer intervention period, on the other hand, may result in aesthetic fatigue and a loss of novelty, with little or no improvement in outcomes. Meanwhile, the results became opposite when removing one study (55) in the effectiveness of 4–8 weeks duration and another (53) in the effectiveness of more than 8 weeks duration. This demonstrates that more well-designed studies of duration are required to evaluate the effectiveness as the current evidence about the effectiveness in duration is not robust. According to the included studies, a single horticulture activity lasted mostly 1–2 h. As Hayashi pointed out, horticultural activities longer than 2 h did not have a positive effect on mood (83). Though previous studies showed that even short-time exercise in gardens provided instantaneous benefits on health, such as reductions in the symptoms of depression and anxiety (65, 83, 84).

**Figure 9:**
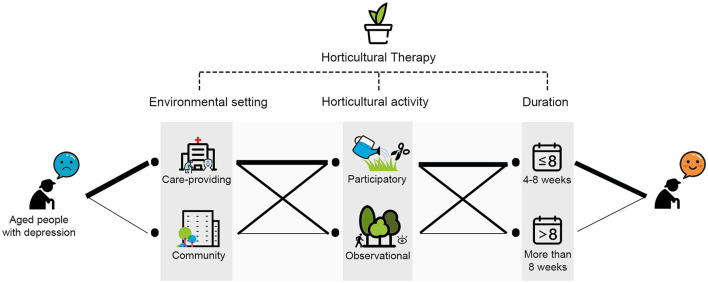
The “prescription pill” of horticultural therapy in the aged people with depression. (Note: thicker lines indicate better therapeutic effect).

We came up with a “prescription pill” of horticultural therapy in the aged people with depression according to our study (as shown in Figure 9).

However, there are some limitations. We only included articles written in English or Chinese, which might result in articles published in other languages not being included in our analysis. And we only classified environmental settings into care-providing and community settings instead of more specific environmental subgroups given the wide range of environments (communities, hospitals, nursing homes, homeless living facility, care centrals and long-term care facilities). Gender of participants and the performers of horticultural therapy (such as researchers, nursing staff, or horticultural therapists) might also influence the outcomes of horticultural therapy on depression reduction in the aged. Therefore, future studies should pay more attention on these issues.

## 5. Conclusion

Our analysis supported the notion that horticultural therapy could improve symptoms in aged people with depression. We also looked into the effectiveness of different interventions of horticultural therapy (including the types of environmental settings, activities, and duration) and then came up with a comprehensive set of recommendations. In terms of the environment settings, care-providing settings were demonstrated to produce higher therapeutic effects. Participatory activities produced greater outcomes than observational activities. In terms of the duration of the interventions, a course of 4–8 weeks of horticultural therapy had better outcomes. Furthermore, future research on horticultural therapy and depression is in high need, and more rigorously designed studies are needed to shed light on which type of environment settings and horticultural interventions leads to improved depression outcomes.

Overall, our results demonstrated that horticultural therapy plays an effective role in promoting the wellbeing of older adults with depression. We also developed a comprehensive framework for future conduct of horticulture therapy (in a methodical and professional manner) based on our subgroup analysis. This research serves as a reference for the planning and implementation of horticultural activities as well as the design of healing gardens. We hope the application of horticultural therapy could help lower national financial expenditures on public health care and bring new ideas and approaches to promote public health. We call on future studies to include additional outcome indicators (such as quality of life, social function) and study designs (such as cross-section study) to demonstrate the efficacy of horticultural therapy for various dimensions of depression, and other diseases as well as groups of people.

## Data availability statement

The original contributions presented in the study are included in the article/Supplementary material, further inquiries can be directed to the corresponding author.

## Author contributions

MX and FX contributed to conception and design of the study. MX, SL, and JL organized the database. MX performed the statistical analysis and wrote the first draft of the manuscript. SL and JL wrote sections of the manuscript. All authors contributed to manuscript revision, read, and approved the submitted version.
