# Supplementary material for: Effectiveness of horticultural therapy in aged people with depression: A systematic review and meta-analysis
Source: Front Public Health. 2023 Mar 8;11:1142456. doi: 10.3389/fpubh.2023.1142456 (PMC10031070; doi:10.3389/fpubh.2023.1142456)
Supplement: Supplementary file 1 [file Table_1.DOCX]

Supplementary Material

Effectiveness of horticultural therapy in aged people with depression: A systematic review and meta-analysis

Meijing Xu†, Shan Lu†, Jianjiao Liu, Feng Xu*

†These authors share first authorship

*** Correspondence:** Corresponding Author: [ccxfcn@sina.com](mailto:ccxfcn@sina.com)

# Supplementary Tables

**Supplementary Table 1. PRISMA 2009 Checklist.**

| ***Section/topic*** | ***#*** | ***Checklist item*** | ***Reported on page #*** |
| --- | --- | --- | --- |
| **TITLE** | | | 1 |
| **Title** | 1 | Identify the report as a systematic review, meta-analysis, or both. | 1 |
| **ABSTRACT** | | | 1 |
| **Structured summary** | 2 | Provide a structured summary including, as applicable: background; objectives; data sources; study eligibility criteria, participants, and interventions; study appraisal and synthesis methods; results; limitations; conclusions and implications of key findings; systematic review registration number. | 1 |
| **INTRODUCTION** | | | 2-3 |
| **Rationale** | 3 | Describe the rationale for the review in the context of what is already known. | 1-2 |
| **Objectives** | 4 | Provide an explicit statement of questions being addressed with reference to participants, interventions, comparisons, outcomes, and study design (PICOS). | 2 |
| **METHODS** | | | 2-4 |
| **Protocol and registration** | 5 | Indicate if a review protocol exists, if and where it can be accessed (e.g., Web address), and, if available, provide registration information including registration number. | 2 |
| **Eligibility criteria** | 6 | Specify study characteristics (e.g., PICOS, length of follow-up) and report characteristics (e.g., years considered, language, publication status) used as criteria for eligibility, giving rationale. | 2 |
| **Information sources** | 7 | Describe all information sources (e.g., databases with dates of coverage, contact with study authors to identify additional studies) in the search and date last searched. | 2 |
| **Search** | 8 | Present full electronic search strategy for at least one database, including any limits used, such that it could be repeated. | 2 |
| **Study selection** | 9 | State the process for selecting studies (i.e., screening, eligibility, included in systematic review, and, if applicable, included in the meta-analysis). | 3 |
| **Data collection process** | 10 | Describe method of data extraction from reports (e.g., piloted forms, independently, in duplicate) and any processes for obtaining and confirming data from investigators. | 3 |
| **Data items** | 11 | List and define all variables for which data were sought (e.g., PICOS, funding sources) and any assumptions and simplifications made. | 3 |
| **Risk of bias in individual studies** | 12 | Describe methods used for assessing risk of bias of individual studies (including specification of whether this was done at the study or outcome level), and how this information is to be used in any data synthesis. | 3-4 |
| **Summary measures** | 13 | State the principal summary measures (e.g., risk ratio, difference in means). | 4 |
| **Synthesis of results** | 14 | Describe the methods of handling data and combining results of studies, if done, including measures of consistency (e.g., I^2^) for each meta-analysis. | 4 |
| **Risk of bias across studies** | 15 | Specify any assessment of risk of bias that may affect the cumulative evidence (e.g., publication bias, selective reporting within studies). | 4 |
| **Additional analyses** | 16 | Describe methods of additional analyses (e.g., sensitivity or subgroup analyses, meta-regression), if done, indicating which were pre-specified. | 4 |
| **RESULTS** | | | 4-6 |
| **Study selection** | 17 | Give numbers of studies screened, assessed for eligibility, and included in the review, with reasons for exclusions at each stage, ideally with a flow diagram. | 4 |
| **Study characteristics** | 18 | For each study, present characteristics for which data were extracted (e.g., study size, PICOS, follow-up period) and provide the citations. | 4-5 |
| **Risk of bias within studies** | 19 | Present data on risk of bias of each study and, if available, any outcome level assessment (see item 12). | 5 |
| **Results of individual studies** | 20 | For all outcomes considered (benefits or harms), present, for each study: (a) simple summary data for each intervention group (b) effect estimates and confidence intervals, ideally with a forest plot. | 5-6 |
| **Synthesis of results** | 21 | Present results of each meta-analysis done, including confidence intervals and measures of consistency. | 5-6 |
| **Risk of bias across studies** | 22 | Present results of any assessment of risk of bias across studies (see Item 15). | 6 |
| **Additional analysis** | 23 | Give results of additional analyses, if done (e.g., sensitivity or subgroup analyses, meta-regression [see Item 16]). | 5-6 |
| **DISCUSSION** | | | 6-9 |
| **Summary of evidence** | 24 | Summarize the main findings including the strength of evidence for each main outcome; consider their relevance to key groups (e.g., healthcare providers, users, and policy makers). | 6-8 |
| **Limitations** | 25 | Discuss limitations at study and outcome level (e.g., risk of bias), and at review-level (e.g., incomplete retrieval of identified research, reporting bias). | 9 |
| **Conclusions** | 26 | Provide a general interpretation of the results in the context of other evidence, and implications for future research. | 9 |
| **FUNDING** | | | 9-10 |
| **Funding** | 27 | Describe sources of funding for the systematic review and other support (e.g., supply of data); role of funders for the systematic review. | 9-10 |

**Supplementary Table 2. Detailed Search Strategy and Results of PubMed.**

| **PubMed search strategy – last searched on September 25, 2022** | | **Results** |
| --- | --- | --- |
| #1 | Search: "Aged"[Mesh] Sort by: Most Recent | [3,415,705](https://pubmed.ncbi.nlm.nih.gov/?sort=date&term=%22Aged%22%5BMesh%5D) |
| #2 | Search: ((elderly) OR ("old people")) OR (older) | [6,095,591](https://pubmed.ncbi.nlm.nih.gov/?term=%28%28elderly%29+OR+%28%22old+people%22%29%29+OR+%28older%29&sort=) |
| #3 | Search: #1 or #2 | [6,095,591](https://pubmed.ncbi.nlm.nih.gov/?term=%28%28elderly%29+OR+%28%22old+people%22%29%29+OR+%28older%29&sort=) |
| #4 | Search: ((melancholia*) OR ("depressive disorder")) OR (depress*) | [623,600](https://pubmed.ncbi.nlm.nih.gov/?term=%28%28melancholia%2A%29+OR+%28%22depressive+disorder%22%29%29+OR+%28depress%2A%29&sort=) |
| #5 | Search: ((horticult*) OR (garden*)) OR (farm*) | [368,257](https://pubmed.ncbi.nlm.nih.gov/?term=%28%28horticult%2A%29+OR+%28garden%2A%29%29+OR+%28farm%2A%29&sort=) |
| #6 | Search: #3 AND #4 AND #5 | [1543](https://pubmed.ncbi.nlm.nih.gov/?term=%235+AND+%233+AND+%232&sort=) |

**Supplementary Table 3. Detailed Search Strategy and Results of Embase.**

| **Embase search strategy – last searched on September 25, 2022** | | **Results** |
| --- | --- | --- |
| #1 | 'aged'/exp OR elderly:ti,ab,kw OR 'old people':ti,ab,kw OR older:ti,ab,kw | 40545547 |
| #2 | melancholia*:ti,ab,kw OR 'depressive disorder':ti,ab,kw OR depress*:ti,ab,kw | [729788](https://www.embase.com/) |
| #3 | horticult* OR garden* OR farm* | 141838 |
| #4 | #1 AND #2 AND #3 | 277 |

**Supplementary Table 4.** **Detailed Search Strategy and Results of The Cochrane Library.**

| **The Cochrane library search strategy – last searched on September 25, 2022** | | **Results** |
| --- | --- | --- |
| #1 | MeSH descriptor: [Aged] explode all trees | 221105 |
| #2 | (elderly):ti,ab,kw OR ("old people"):ti,ab,kw OR (older):ti,ab,kw (Word variations have been searched) | 111309 |
| #3 | (melancholia*):ti,ab,kw OR ("depressive disorder"):ti,ab,kw OR (depress*):ti,ab,kw (Word variations have been searched) | 100230 |
| #4 | (horticult*):ti,ab,kw OR (garden*):ti,ab,kw OR (farm*):ti,ab,kw (Word variations have been searched) | 6470 |
| #5 | #1 OR #2 | 304949 |
| #6 | #3 AND #4 AND #5 | 140 |

**Note:** 84 Cochrane reviews, 56 trails

**Supplementary Table 5. Detailed Search Strategy and Results of Medline.**

| **Medline search strategy – last searched on September 25, 2022** | | **Results** |
| --- | --- | --- |
| 1 | aged (MeSH 主题词) or elderly (主题) or "old people" (主题) or older (主题) | 4697945 |
| 2 | melancholia* (主题) or "depressive disorder" (主题) or depress* (主题) | 619674 |
| 3 | horticult* (主题) or garden* (主题) or farm* (主题) | 147889 |
| 4 | #3 AND #2 AND #1 | 423 |

**Supplementary Table 6. Detailed Search Strategy and Results of CINAHL.**

| **CINAHL search strategy – last searched on September 25, 2022** | | **Results** |
| --- | --- | --- |
| S1 | aged OR elderly OR "old people" OR older | 1189634 |
| S2 | melancholia* OR "depressive disorder" OR depress* | 216888 |
| S3 | horticult* OR garden* OR farm* | 23847 |
| S4 | (horticult* OR garden* OR farm*) AND (S1 AND S2 AND S3) | 218 |

**Supplementary Table 7.** **Detailed Search Strategy and Results of PsycINFO.**

| **psycINFO search strategy – last searched on September 25, 2022** | | **Results** |
| --- | --- | --- |
| S1 | TI aged OR TI elderly OR TI "old people" OR TI older | 88381 |
| S2 | AB aged OR AB elderly OR AB "old people" OR AB older | [438682](https://www.embase.com/) |
| S3 | TI melancholia* OR TI "depressive disorder" OR TI depress* | 127738 |
| S4 | AB melancholia* OR AB "depressive disorder" OR AB depress* | 332927 |
| S5 | TI horticult* OR TI garden* OR TI farm* | 4043 |
| S6 | AB horticult* OR AB garden* OR AB farm* | 11391 |
| S7 | S1 OR S2 | 449825 |
| S8 | S3 OR S4 | 338401 |
| S9 | S5 OR S6 | 12503 |
| S10 | S7 AND S8 AND S9 | 130 |

**Supplementary Table 8. Detailed Search Strategy and Results of Web of Science.**

| **Web of Science search strategy – last searched on September 25, 2022** | | **Results** |
| --- | --- | --- |
| 1 | ((ALL=(melancholia*)) OR ALL=("depressive disorder")) OR ALL=(depress*) | [733,595](https://www.webofscience.com/wos/woscc/summary/ccea69d5-2983-4644-916f-e49e5f05ad20-51511271/relevance/1) |
| 2 | (((ALL=(aged)) OR ALL=(elderly)) OR ALL=("old people")) OR ALL=(older) | [4,717,266](https://www.webofscience.com/wos/woscc/summary/85ca5e00-8d86-44dc-9828-3f43896b361d-5151380c/relevance/1) |
| 3 | ((ALL=(horticult*)) OR ALL=(garden*)) OR ALL=(farm*) | [995,795](https://www.webofscience.com/wos/woscc/summary/b86d8caf-2355-4d39-8b90-0faeae05c825-515170da/relevance/1) |
| 4 | #3 AND #2 AND #1 | [2,169](https://www.webofscience.com/wos/woscc/summary/590c47b7-be1a-44b1-bc97-078822c0c3fb-51517b57/relevance/1) |

**Supplementary Table 9. Detailed Search Strategy and Results of Scopus.**

| **Scopus search strategy – last searched on September 25, 2022** | | **Results** |
| --- | --- | --- |
| 1 | (TITLE-ABS-KEY(aged) OR TITLE-ABS-KEY (elderly) OR TITLE-ABS-KEY("old people") OR TITLE-ABS-KEY ( older ) ) | 7718647 |
| 2 | (TITLE-ABS-KEY(melancholia*) OR TITLE-ABS-KEY("depressive disorder") OR TITLE-ABS-KEY(depress*)) | 1012479 |
| 3 | (TITLE-ABS-KEY(horticult*) OR TITLE-ABS-KEY(garden*) OR TITLE-ABS-KEY(farm*)) | 629089 |
| 4 | ((TITLE-ABS-KEY(aged) OR TITLE-ABS-KEY(elderly) OR TITLE-ABS-KEY("old people") OR TITLE-ABS-KEY(older))) AND ((TITLE-ABS-KEY(melancholia*) OR TITLE-ABS-KEY("depressive disorder") OR TITLE-ABS-KEY(depress*))) AND ((TITLE-ABS-KEY(horticult*) OR TITLE-ABS-KEY(garden*) OR TITLE-ABS-KEY(farm*))) | 997 |

**Supplementary Table 10. Detailed Search Strategy and Results of ProQuest.**

| **ProQuest search strategy – last searched on September 25, 2022** | | **Results** |
| --- | --- | --- |
| S1 | (su(aged) OR su(elderly) OR su("old people") OR su(older)) AND stype.exact("Scholarly Journals") | 356003 |
| S2 | (su(melancholia*) OR su("depressive disorder") OR su(depress*)) AND stype.exact("Scholarly Journals") | 119979 |
| S3 | (su(horticult*) OR su(garden*) OR su(farm*)) AND stype.exact("Scholarly Journals") | 131508 |
| S4 | S1 AND S2 AND S3 | 41 |

**Supplementary Table 11. Detailed Search Strategy and Results of CNKI.**

| **CNKI search strategy – last searched on September 25, 2022** | | **Results** |
| --- | --- | --- |
| #1 | 主题=老人OR主题=老年OR主题=老龄OR主题=长者 | 408975 |
| #2 | 主题=抑郁OR主题=忧郁 | [129001](https://www.embase.com/) |
| #3 | 主题=花园 OR主题=园艺 OR主题=农 | 311893 |
| #4 | #1 AND #2 AND #3 | 7 |

**Supplementary Table 12. Detailed Search Strategy and Results of Wanfang Dat**a**.**

| **Wanfang Data search strategy – last searched on September 25, 2022** | | **Results** |
| --- | --- | --- |
| #1 | 主题:("老人") or 主题:("老年") or 主题:("老龄") or 主题:("长者") | 981953 |
| #2 | 主题:("抑郁") or 主题:("忧郁") | [253123](https://www.embase.com/) |
| #3 | 主题:("花园") or 主题:("园艺") or 主题:("农") | 3800479 |
| #4 | #1 AND #2 AND #3 | 527 |

**Supplementary Table 13. Detailed Search Strategy and Results of VIP Data.**

| **VIP Datasearch strategy – last searched on September 25, 2022** | | **Results** |
| --- | --- | --- |
| 1# | [(((任意字段=老人 OR 任意字段=老年) OR 任意字段=老龄) OR 任意字段=长者)](http://lib.cqvip.com/Qikan/search/index?LngMySearHistoryIdGuid=bcd93e20-11b7-4b90-9d8a-1990b5fb5d7c&from=Qikan_Article_History) (精确) | 827511 |
| 2# | [(任意字段=抑郁 OR 任意字段=忧郁)](http://lib.cqvip.com/Qikan/search/index?LngMySearHistoryIdGuid=074de22a-88da-497f-aa18-cc0c7347b5e3&from=Qikan_Article_History) (精确) | [202438](https://www.embase.com/) |
| 3# | [((任意字段=花园 OR 任意字段=园艺) OR 任意字段=农)](http://lib.cqvip.com/Qikan/search/index?LngMySearHistoryIdGuid=4888c844-a60a-4a50-bc70-6783a26d5dae&from=Qikan_Article_History) (精确) | 1881024 |
| 4# | 1# AND 2# AND 3# | 276 |

**Supplementary Table 14. Detailed Search Strategy and Results of CBM.**

| **CBM search strategy – last searched on September 25, 2022** | | **Results** |
| --- | --- | --- |
| #1 | "老人"[全部字段:智能] OR "老年"[全部字段:智能] OR "老龄"[全部字段:智能] OR "长者"[全部字段:智能] | 702095 |
| #2 | "抑郁"[全部字段:智能] OR "忧郁"[全部字段:智能] | [167965](https://www.embase.com/) |
| #3 | "花园"[全部字段:智能] OR "园艺"[全部字段:智能] OR "农"[全部字段:智能] | 377714 |
| #4 | 1# AND 2# AND 3# | 618 |

**Supplementary Table 15. Main characteristics of the selected studies.**

| Author (Publication Year; Country) | Study design | Setting | Subject | | | Intervention | | | | | Measurement and Outcome |
| --- | --- | --- | --- | --- | --- | --- | --- | --- | --- | --- | --- |
|  |  |  | Participants E/C | Age E/C | Male (%) | Performer | Frequency and duration | Follow-up | Intervention-E | Intervention-C |  |
| Chen, Yuh-Min and Ji, Jeng-Yi (2015; China) | Quasi-experimental study | Nursing home | 10 | 75.3 | 60 | - | 90 min per time/once per week/10 weeks | 5 and 10 weeks | Participatory horticultural activities in reality: planting green bean seeds, decorating flowerpots，arranging cut flowers, etc. | None | GDS-15 |
| Chu Hui-Ying et al. (2019; China) | RCT | Nursing Home | 75/75 | 79.2/77.9 | 33.3/41.3 | A lead researcher and four research assistants who had horticulture licenses | 90-120 min per time/once per week/8 weeks | 8 weeks | Participatory horticultural activities in reality: watering plant, making grass doll, drawing with dry flowers and leaves, etc. | Leisure activities (e.g. watching TV, listening to music) | GDS-15 |
| Gu Wen-yun (2020; China) | Quasi-experimental study | Community | 22 | 79.0 | 29.2 | - | 60 min per time/twice per week/12 weeks | 4, 8, and 12 weeks | Participatory horticultural activities in reality: making potted plants, managing hydroponic plants, pruning and cutting Chinese rose, etc. | None | GDS-30 |
| Jiang Na et al. (2022; China) | RCT | Nursing home | 30/30 | ≥60/≥60 | 40.0/46.7 | A horticulturist, a rehabilitation therapist | 90 min per time/ once per week/8 weeks | 8 weeks | Participatory horticultural activities in reality: taking care of potted plants, planting vegetable seeds, designing "mini garden", etc. | Routine nursing | GDS-30 |
| Kim, Y. H. et al.^a^ (2020; Korea) | Quasi-experimental study | Homeless Living Facility | 6/6 | 72.4/74.0 | 66.7/66.7 | A facilitator and 3 assistant facilitators of doctor’s and master’s program in horticultural therapy | 60 to 90 min per time/ once per week/12 weeks | 12 weeks | Participatory horticultural activities in reality: managing the garden (watering, removing weeds, thinning out, etc.) | None | GDS-15 |
| Kim, Y. H. et al.^b1^ (2020; Korea) | Quasi-experimental study | Care facility: health center | 13 | 81.7 | 0 | A registered horticultural therapist and 2 assistants | 100 min per time/once per week/15 weeks | 15 weeks | Participatory horticultural activities in reality: making pressed flower name tags, making grass dolls, flower arrangement, harvesting and cooking sprout vegetables, etc. | None. | GDS-15 |
| Kim, Y. H. et al.^b2^ (2020; Korea) | Quasi-experimental study | Care facility: health center | 8 | 81.9 | 0 | A registered horticultural therapist and 2 assistants | 100 min per time/twice per week/8 weeks | 8 weeks | Participatory horticultural activities in reality: making pressed flower name tags, making grass dolls, flower arrangement, harvesting and cooking sprout vegetables, etc. | None. | GDS-15 |
| Makizako Hyuma et al. (2020; Japan) | RCT | Community | 30/59 | 73.1/73.1 | 53.3/52.5 | Vegetable experts | 60-90 min per time/once per week/20 weeks | 20 and 48 weeks | Participatory horticultural activities in reality: cultivating, growing, harvesting, and group planting | An exercise program; education classes | GDS-15 |
| McCaffrey, R. et al. (2010; Japan) | Quasi-experimental design | Community | 40 | 71.3 | - | Researcher | 120 min per time/12 times in total/36 weeks | 36 weeks | Observational horticultural activities in reality: walking the garden with themes | None | GDS-15 |
| McCaffrey Ruth et al. (2011; Japan) | RCT | Community | 26/13 | 74.6/74.3 | 38.5/46.2 | Researchers | 60 to 120 min per time /twice per week/6 weeks | 6 weeks | Observational horticultural activities in reality: walking through the garden with guided imagery or without walking guidance | Art therapy | GDS-30 |
| Szczepanska-Gieracha, J. et al. (2021; Poland) | RCT | Care facility: foundation for Senior Citizen | 11/12 | 70.2/71.3 | 0/0 | A physiotherapist and a psychotherapist | 20 min per time/ twice per week/4 weeks | 4 and 6 weeks | Virtual and participatory horticultural activities: coloring the mandala with appropriate colors in the central place of virtual garden | General fitness training and psychoeducation | GDS-30 |
| Tsung-Yi, Lin. et al. (2020; China) | Quasi-experimental study | Long-term care facility | 59/47 | 77.4/78.4 | 81/53 | A facilitator and some graduate students who were trained to acquired basic horticultural therapy skills | 60 min per time/ twice per week/9 weeks | 9 and 17 weeks | Virtual and participatory horticultural activities: cultivating plants, creating seeds puzzles, designing potted plants, etc. | Long-term care | GDS-15 |
| Wang Zhen-lan and Jiang Ding-wu (2020; China) | RCT | Hospital | 63/63 | 67.9/68.6 | 42.9/44.4 | - | 60-120 min per time/six times per week/- | - | Participatory horticultural activities in reality: planting flowers, vegetables, fruits, and other plants; making potted plants, flower arrangements, etc. | Sports Care | GDS-30 |

Abbreviations: E for Experimental; C for Control; GDS-15 for the 15-item Geriatric Depression Scale; GDS-30 for the 30-item Geriatric Depression Scale.

*Note:* a and b indicate that they are two studies from the same year; b1 and b2 indicate that they are two reports from the same study (Kim, Y. H. et al.^b^, 2020).
